# Increased Cardiovascular Reactivity to Acute Stress and Salt-Loading in Adult Male Offspring of Fat Fed Non-Obese Rats

**DOI:** 10.1371/journal.pone.0025250

**Published:** 2011-10-17

**Authors:** Olena Rudyk, Péter Makra, Eugene Jansen, Michael J. Shattock, Lucilla Poston, Paul D. Taylor

**Affiliations:** 1 Division of Women's Health, School of Medicine, King's College London, London, United Kingdom; 2 Cardiovascular Division, King's College London, London, United Kingdom; 3 Department of Experimental Physics, University of Szeged, Szeged, Hungary; 4 Laboratory for Health Protection Research, National Institute for Public Health and the Environment, Bilthoven, The Netherlands; Pennington Biomedical Research Center, United States of America

## Abstract

Diet-induced obesity in rat pregnancy has been shown previously to be associated with consistently raised blood pressure in the offspring, attributed to sympathetic over-activation, but the relative contributions to this phenotype of maternal obesity *versus* raised dietary fat is unknown. Sprague-Dawley female rats were fed either a control (4.3% fat, n = 11) or lard-enriched (23.6% fat, n = 16) chow 10 days prior to mating, throughout pregnancy and lactation. In conscious adult (9-month-old) offspring cardiovascular parameters were measured (radiotelemetry). The short period of fat-feeding did not increase maternal weight *versus* controls and the baseline blood pressure was similar in offspring of fat fed dams (OF) and controls (OC). However, adult male OF showed heightened cardiovascular reactivity to acute restraint stress (p<0.01; Δ systolic blood pressure (SBP) and Δheart rate (HR)) with a prolonged recovery time compared to male OC. α1/β-adrenergic receptor blockade normalised the response. Also, after dietary salt-loading (8%-NaCl *ad libitum* for 1 week) male OF demonstrated higher SBP (p<0.05) in the awake phase (night-time) and increased low/high frequency ratio of power spectral density of HR variability *versus* OC. Baroreflex gain and basal power spectral density components of the heart rate or blood pressure were similar in male OF and OC. Minor abnormalities were evident in female OF. Fat feeding in the absence of maternal obesity in pregnant rats leads to altered sympathetic control of cardiovascular function in adult male offspring, and hypertension in response to stressor stimuli.

## Introduction

Growing evidence suggests that the worldwide prevalence of obesity amongst pregnant women and the abundance of calorie rich diets may contribute to heightened cardiovascular and metabolic risk in the child. Several investigations in mother-child cohorts have indicated that adiposity in children is independently associated with maternal obesity or maternal gestational weight gain [1], [2], [3] and studies of experimental animals fed calorie rich diets provide convincing evidence for maternal ‘transmission’ of obesity and cardiovascular dysfunction to the offspring [4], [5], [6]. Despite being reared on a standard chow diet, offspring of rodents fed fat (lard) or highly palatable (lard and sugar enriched) diets develop insulin resistance, and in adulthood are predisposed to obesity [4], [5], [7], [8].

Most recently, in a study of maternal diet-induced obesity, several related observations including altered heart rate variability (HRV) as well as abnormal baroreceptor responsiveness, and hypertension in the offspring led us to conclude that aberrant autonomic control of cardiovascular function could be acquired in the earliest stages of life [8]. An understanding of the relative contribution of obesity and diet to development of this sympathetic over-activity and associated hypertension could contribute to development of dietary interventions in pregnant women to optimise cardiovascular health of the offspring. Others have suggested that maternal obesity, rather than the high fat diet *per se*, is a pre-requisite for development of offspring obesity, hyperleptinaemia [6], [9] and insulin resistance [6] in rats. However, offspring cardiovascular variables were not determined [6].

In the present study we have investigated the specific influence of a high fat diet on offspring autonomic function in the absence of overt maternal obesity. To achieve this, female rats were fed a diet enriched with animal lard for a limited period prior to pregnancy and during pregnancy and lactation. We investigated the autonomic responsiveness of blood pressure and heart rate (HR) to the challenges of acute stress [10] and salt-loading [11] in adult (9-month-old) conscious offspring employing remote radio-telemetric recording. Adrenergic receptor blockade was employed to define the role of the sympathetic nervous system in the cardiovascular response to immobilisation stress and salt-loading. Basal baroreceptor function was also analysed. In addition, HRV and systolic blood pressure variability (BPV) parameters were determined as a measure of autonomic function [12], [13].

## Results

### Maternal Characteristics

**Figure 1:**
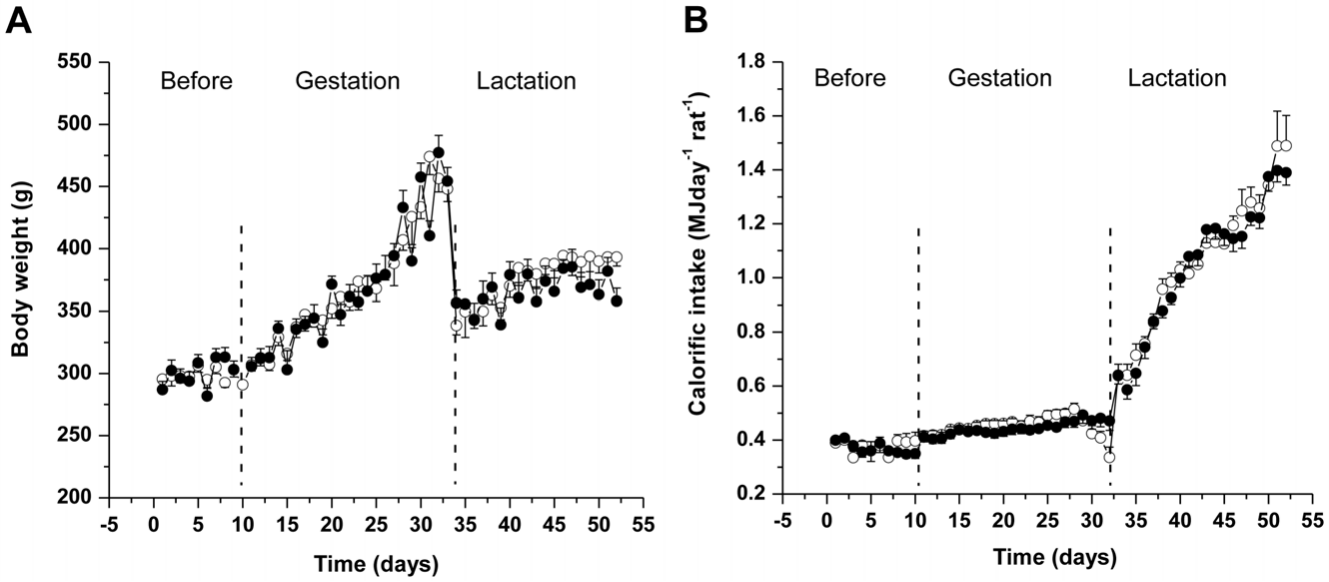
Control and fat-fed dams had a similar body weight gain and calorific intake. (A) Body weight, g, and (B) calorific intake, MJ rat^−1^ day^−1^, in dams fed a control (open circles) or a fat diet (closed circles), n = 11 for control, n = 16 for fat-fed dams. Error bars represent mean±SEM.

BW during the pre-conception and gestation periods was similar in all dams (Figure 1). Fat fed dams consumed less food by weight compared to dams fed the standard chow; such that calorific intake was similar between the groups (Figure 1 and Table 1). At day 21 postpartum there were no differences in maternal plasma leptin (control dams: 1.44±0.16 ng ml^−1^ *versus* fat fed dams: 1.50±0.10 ng ml^−1^, p = 0.60, n = 5–8) or insulin (control dams: 343±107 pmol l^−1^ *versus* fat fed dams: 308±50 pmol l^−1^, p = 0.74, n = 5–8) concentration between groups. Litter size was not different between control and fat fed dams.

**Table 1:** Content of fatty acids in experimental diets.

Fatty acids	Palmitic (SFA)	Stearic (SFA)	Palmitoleic (MUFA)	Oleic (MUFA)	Linoleic (PUFA)	Linolenic (PUFA)	Arachidonic (PUFA)
Control diet (%)	0.36	0.08	0.13	1.03	1.15	0.17	0.22
LARD-rich diet (%)	4.52	2.00	0.12	6.92	2.63	0.24	0.18

### Offspring Plasma Parameters

At 9 months of age, male offspring (OC: 684±17 g *versus* OF: 726±24 g, p = 0.19, n = 9–11) and female offspring (OC: 398±17 g *versus* OF: 399±12 g, p = 0.90, n = 11–15) were of similar weight and there were no differences in abdominal fat mass (pooled retroperitoneal, gonadal and perirenal fat pads) or fat pad mass/BW ratio. Leptin (OC: 7.70±1.40 ng ml^−1^ *versus* OF: 8.50±0.80 ng ml^−1^, p = 0.65, n = 6–8) and insulin (OC: 743±210 pmol l^−1^ *versus* OF: 986±123 pmol l^−1^, p = 0.30, n = 6–8) concentrations were similar between OF and OC males whereas OF females were hypoleptinaemic compared with OC (OC: 8.00±1.90 ng ml^−1^ *versus* OF: 2.90±0.60 ng ml^−1^, p = 0.01, n = 6–8) and the plasma insulin concentration was comparable (OC: 636±153 pmol l^−1^ *versus* OF: 799±116 pmol l^−1^, p = 0.40, n = 6–8). There were no significant differences in plasma free fatty acids (FFA) (OC: 0.29±0.04 mmol l^−1^ *versus* OF: 0.4±0.05 mmol l^−1^, p = 0.10, n = 6–8) concentrations between OF and OC males whereas OF females demonstrated significantly higher FFA (OC: 0.25±0.02 mmol l^−1^ *versus* OF: 0.57±0.1 mmol l^−1^, p = 0.02, n = 6–8) concentrations compared with OC. Basal plasma noradrenaline concentration was similar between OF and OC male offspring (OC: 1.25±0.16 ng ml^−1^ *versus* OF: 0.96±0.2 ng ml^−1^, p = 0.30, n = 6–8) although it was significantly elevated in OF females (OC: 0.84±0.07 ng ml^−1^ *versus* OF: 1.08±0.08 ng ml^−1^, p = 0.04, n = 6–8).

### Offspring Cardiovascular Parameters

Baseline SBP, DBP, MAP, HR and locomotor activity were comparable between OC and OF male and female progeny. DBP and MAP were lower and HR was higher in OC females compared to OC males (Table 2, and Tables S1 and S2) whereas HR and locomotor activity were higher in OF females compared to OF males.

**Table 2:** Basal cardiovascular parameters and activity in adult offspring born to dams fed a control (OC) or a fat diet (OF).

	OC	OF	OC	OF
	(n = 11) (n = 11)	(n = 11) (n = 15)
	Males		Females	
Day Systolic blood pressure (mmHg)	126.7±2.7 (8.9)	128.8±1.4 (4.6)	121.6±2.0 (6.6)	124.1±2.1 (8.1)
Night Systolic blood pressure (mmHg)	129.7±2.8 (9.3)	131.7±1.7 (5.6)	125.2±2.1 (7.0)	127.4±2.3 (8.9)
Day Diastolic blood pressure (mmHg)	90.7±1.8 (6.0)	86.3±1.5 (5.0)	82.1±1.4 (4.6)††	83.8±1.1 (4.3)
Night Diastolic blood pressure (mmHg)	92.9±1.6 (5.3)	89.1±1.9 (6.3)	84.3±1.3 (4.3)††	85.9±1.1 (4.3)
Day Heart Rate (bpm)	330±5 (17)	323±5 (17)	354±7 (23)†	367±7 (27)††
Night Heart Rate (bpm)	347±4 (13)	341±4 (13)	377±6 (20)††	388±6 (23)††
Day Mean arterial pressure (mmHg)	106.6±1.6 (5.3)	104.5±1.3 (4.3)	100.0±1.4 (4.6)††	102.0±1.4 (5.4)
Night Mean arterial pressure (mmHg)	109.1±1.5 (5.0)	106.8±1.5 (5.0)	102.0±1.4 (4.6)††	104.5±1.5 (5.8)
Day Activity (counts per min)	1.96±0.16 (0.53)	1.64±0.12 (0.4)	2.15±0.22 (0.73)	2.50±0.14 (0.54)††
Night Activity (counts per min)	2.55±0.13 (0.43)	2.27±0.18 (0.6)	3.00±0.22 (0.73)	3.48±0.22 (0.85)††

Data given as mean±SEM (SD).

† P≤0.05,

†† P≤0.01 *versus* offspring of the same dietary group.

### Baseline autonomic function and baroreflex sensitivity

Time-domain parameters of the basal HRV and power spectral density of both HRV and BPV for all groups are presented in Table 3. In male and female progeny, no differences were observed between OF and OC in baseline time domain parameters and frequency domain parameters of the HRV and BPV. Mean interbeat interval (IBI) was lower in OC females than in OC males.

**Table 3:** Heart rate and blood pressure variability parameters in adult offspring born to dams fed a control (OC) or a fat diet (OF).

	OC	OF	OC	OF
	(n = 11)(n = 11)	(n = 11)(n = 15)
	Males		Females	
**Time domain parameters**				
Mean IBI, ms	203±6 (20.0)	205±3 (10.0)	90±4 (13)†††	185±5 (19)
SD IBI, ms	7.7±0.8 (2.7)	7.2±0.7 (2.3)	9.1±0.8 (2.7)	8.0±1.0 (3.9)
RMSSD	4.7±0.5 (1.7)	3.9±0.3 (1.0)	5.8±0.7 (2.3)	4.6±0.7 (2.7)
**Frequency domain parameters**				
LF, ms^2^	3.2±0.5 (1.7)	2.6±0.5 (1.7)	5.1±1.3 (4.3)	3.1±0.6 (2.3)
HF, ms^2^	8.9±2.1 (7.0)	6.9±1.3 (4.3)	22.7±9.8 (32.5)	10.5±3.3 (12.9)
LF/HF ratio (for HRV)	0.38±0.04 (0.1)	0.46±0.09 (0.3)	0.34±0.03 (0.1)	0.41±0.07 (0.3)
LF, mmHg^2^	2.3±0.6 (2.0)	2.1±0.3 (1.0)	3.0±0.6 (2.0)	2.6±0.3 (1.1)
HF, mmHg^2^	0.7±0.1 (0.3)	0.8±0.1 (0.3)	1.3±0.2 (0.7)†	1.2±0.2 (0.8)
LF/HF ratio (for systolic BPV)	3.80±0.70 (2.3)	3.20±0.50 (1.7)	2.60±0.50 (1.7)	2.70±0.50 (1.9)

IBI, inter-beat interval; SD IBI, standard deviation of inter-beat interval; RMSSD, root mean square of successive differences. Data given as mean±SEM (SD).

† P≤0.05,

††† P≤0.001 *versus* offspring of the same dietary group.

Correlation analysis (using Pearson's product-moment correlation) between maternal weight gain and offspring mean SBP was performed and there was a positive association independent of maternal diet (day-time, resting phase SBP: r = 0.543, r^2^ = 0.295 (95% CI for r^2^ 0.025 to 0.616, p = 0.009); night-time, active phase SBP: r = 0.536, r^2^ = 0.287 (95% CI for r^2^ 0.022 to 0.610), p = 0.010 n = 22).

There were no sex–related differences in baroreflex responses, therefore male and females were pooled for analysis. Maximal (HR, mean±SEM (SD), OC: 469±12 (38) bpm versus OF: 441±11 (35) bpm, p = 0.11, n = 10) and minimal (HR, mean±SEM (SD), OC: 249±19 (60) bpm versus OF: 280±5 (16) bpm, p = 0.15, n = 10) HR responses were similar between OC and OF as was maximal baroreflex gain (mean±SEM (SD),OC: −2.2±0.4 (1.26) bpm/mmHg versus OF: −1.9±0.3 (0.95) bpm/mmHg, p = 0.50, n = 10). Maximal baroreflex gain occurred at MAP, mean±SEM (SD), 92±3 (9) mmHg for OC and 88±4 (13) mmHg for OF respectively.

### Cardiovascular response to acute stress

**Figure 2:**
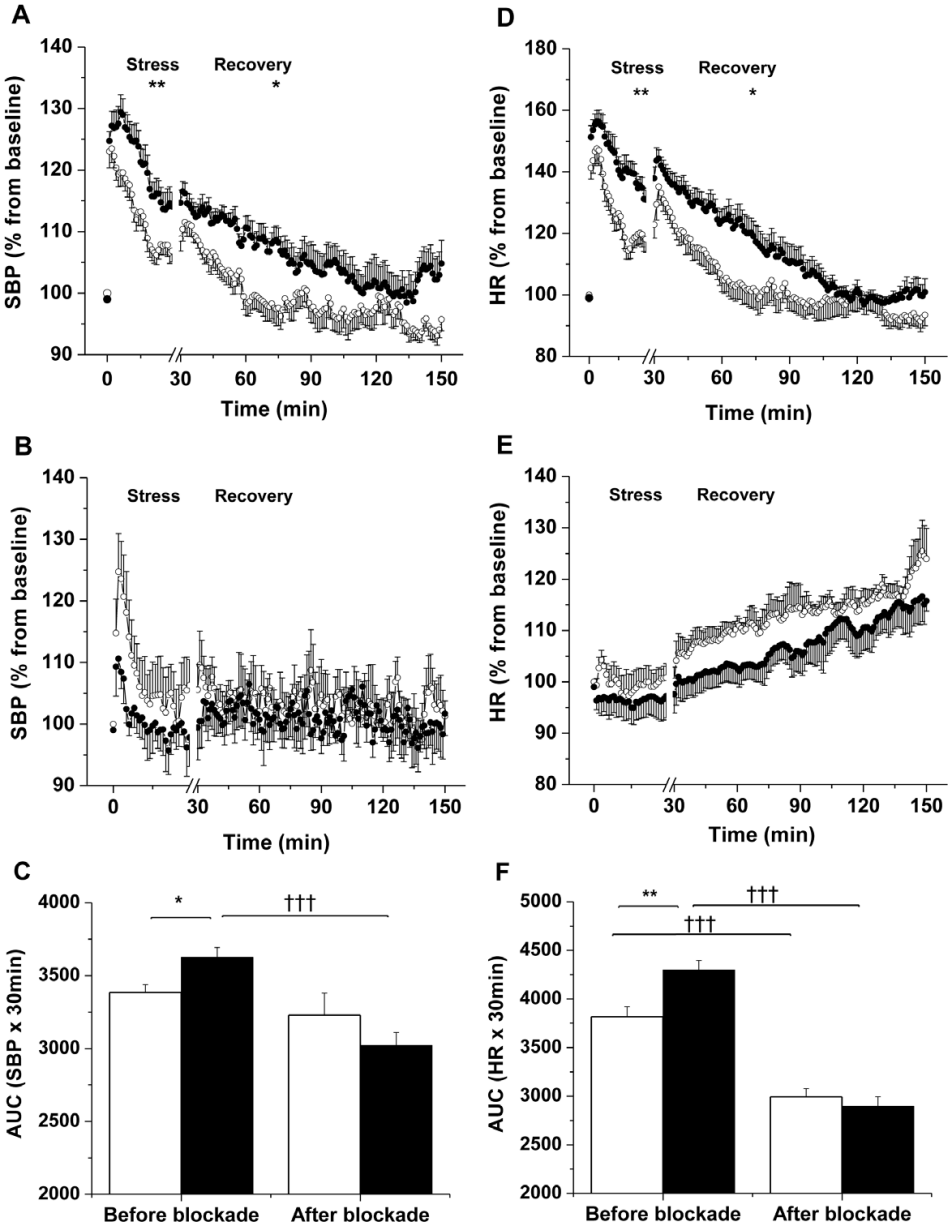
Male offspring of fat-fed dams had an increased pressor and chronotropic response during acute stress. (A, B) SBP and (D, E) HR responses during 30 min of acute stress and 120 min of recovery, before (A, D) and after adrenergic receptor blockade (B, E) in male offspring born to dams fed a control (open circles) or a fat diet (closed circles), n = 11 per group before blockade; n = 5 per group after blockade. *P≤0.05, **P≤0.01 versus control. (C) Area under the curve (AUC) for SBP and (F) HR during stress before and after blockade in male offspring born to dams fed a control (unfilled bars) or a fat diet (filled bars), n = 11 per group before blockade; n = 5 per group after blockade. *P≤0.05, **P≤0.01 versus control. †††P≤0.001 versus before blockade. Error bars represent mean±SEM.

OF males exhibited an exaggerated increase in SBP and HR following restraint stress compared with OC males (SBP: p = 0.009; HR: p = 0.003, n = 11) (Figure 2, and Figures S3 and S4). Recovery from stress was prolonged in OF males with increased SBP (p = 0.02, n = 11) and HR (p = 0.04, n = 11) during the post-stress period compared with OC males, with a slower return to baseline SBP (OC: 32±8 min *versus* OF: 60±10 min, p = 0.04, n = 11). Combined adrenergic receptor blockade prevented stress-induced increase of SBP in OF but not in OC males whereas stress-induced changes of the HR following adrenergic receptor blockade were abolished in both OC and OF males (Figure 2).

**Figure 3:**
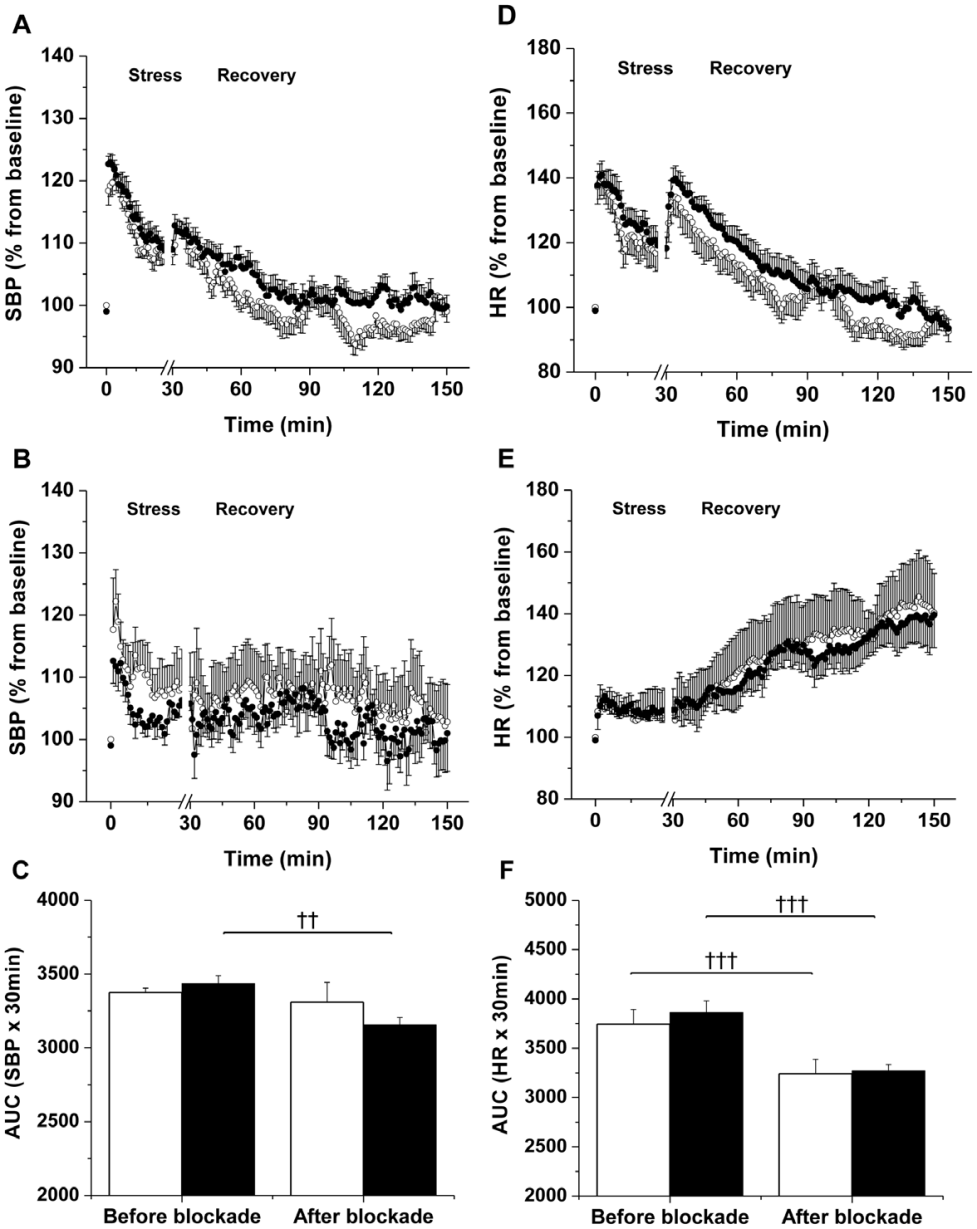
Female offspring of fat-fed dams had a similar stress-response compared with controls. (A, B) SBP and (D, E) HR responses during 30 min of acute stress and 120 min of recovery, before (A, D) and after adrenergic receptor blockade (B, E) in female offspring born to dams fed a control (open circles) or a fat diet (closed circles), n = 5–15 per group. (C) Area under the curve for SBP and (F) HR during stress before and after blockade in female offspring born to dams fed a control (unfilled bars) or a fat diet (filled bars). n = 15 per group before blockade; n = 6 per group after blockade. ††P≤0.01, †††P≤0.001 versus before blockade. Error bars represent mean±SEM.

In females, differences in cardiovascular responses to restraint were not significantly different between OC and OF (SBP: p = 0.36; HR: p = 0.55, n = 11–15) and there was no effect of maternal diet on post-stress recovery (SBP: p = 0.11; HR: p = 0.34, n = 11–15) (Figure 3). However, adrenergic receptor blockade prevented the stress-induced increase of SBP in OF but not in OC females. Stress-induced changes of the HR were abolished after blockade in both OC and OF females (Figure 3).

DBP and MAP showed the same pattern for the stress-response before and after adrenergic blockade as SBP and HR, with OF males being significantly different from male OC littermates (Figures S1 and S2) but no differences were observed between females.

### Cardiovascular response to salt-loading

**Figure 4:**
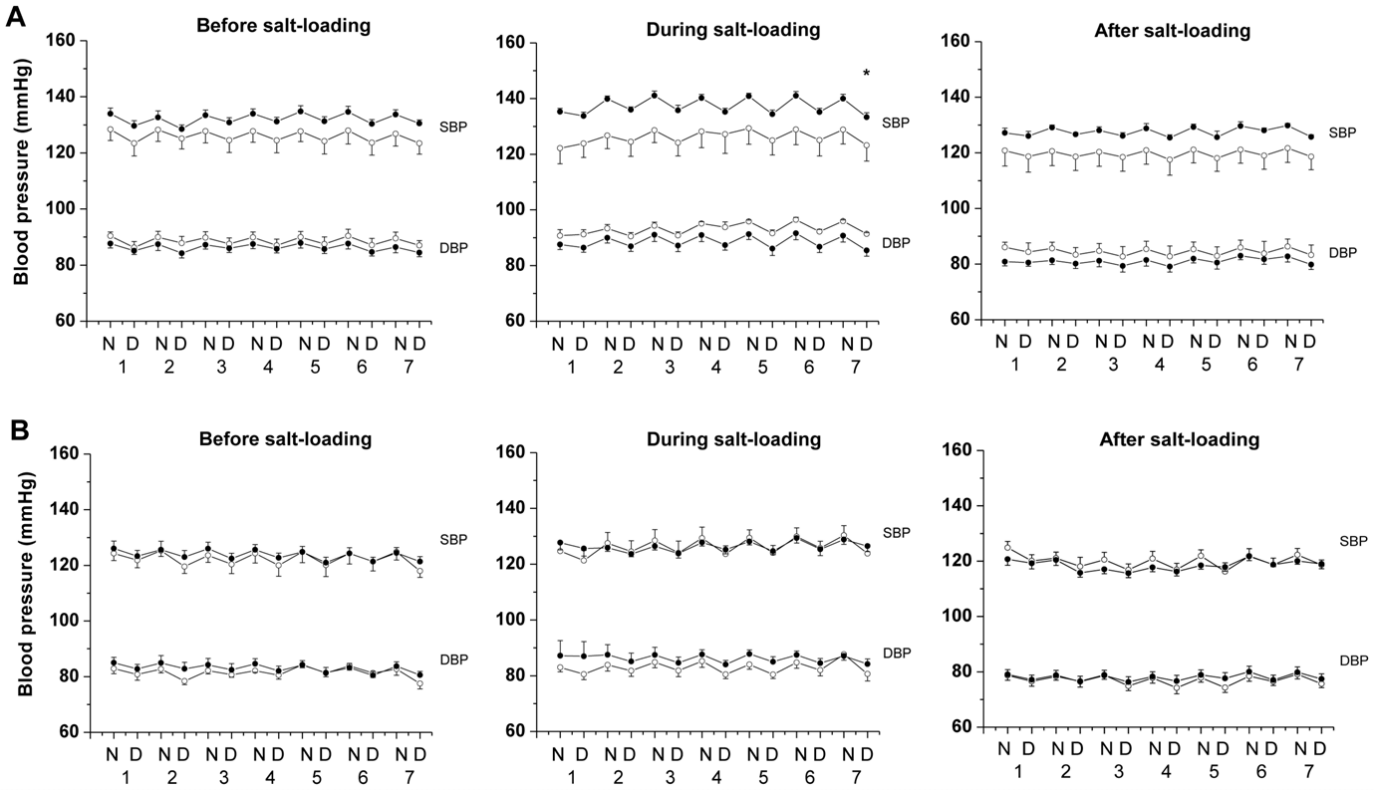
Male offspring of fat-fed dams had an increased pressor response to salt-loading, but not females. SBP and DBP, mmHg, before, during and after salt-loading in (A) male and (B) female offspring of control (open circles) or fat fed dams (closed circles), n = 6 per group. *P<0.05 *versus* control (night-time; active phase). Error bars represent mean±SEM.

**Figure 5:**
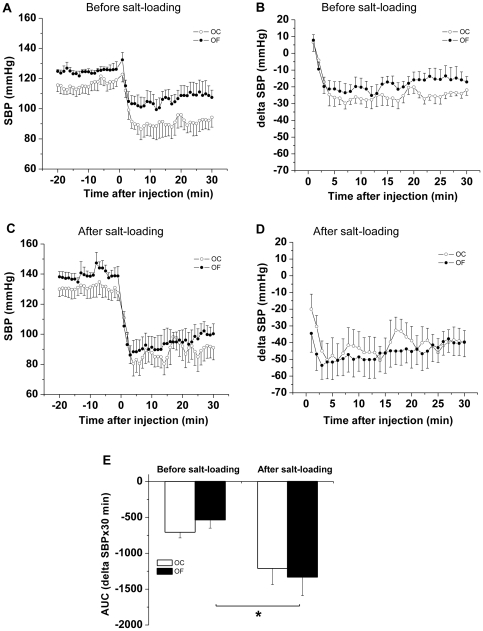
Male offspring of fat-fed dams had a greater depressor response following adrenergic blockade after salt-loading than before. (A, C) SBP response and delta SBP (B, D) in response to combined adrenergic blockade before (A, B) and after (C, D) salt-loading in adult male offspring from dams fed a control (OC, open circles) or a fat diet (OF, closed circles). (E) Area under the curve of delta SBP in response to combined adrenergic blockade before and after salt-loading in adult male offspring from dams fed a control (unfilled bars) or a fat diet (filled bars). n = 5 per group, * P≤0.05 versus before salt-loading). Error bars represent mean±SEM.

**Figure 6:**
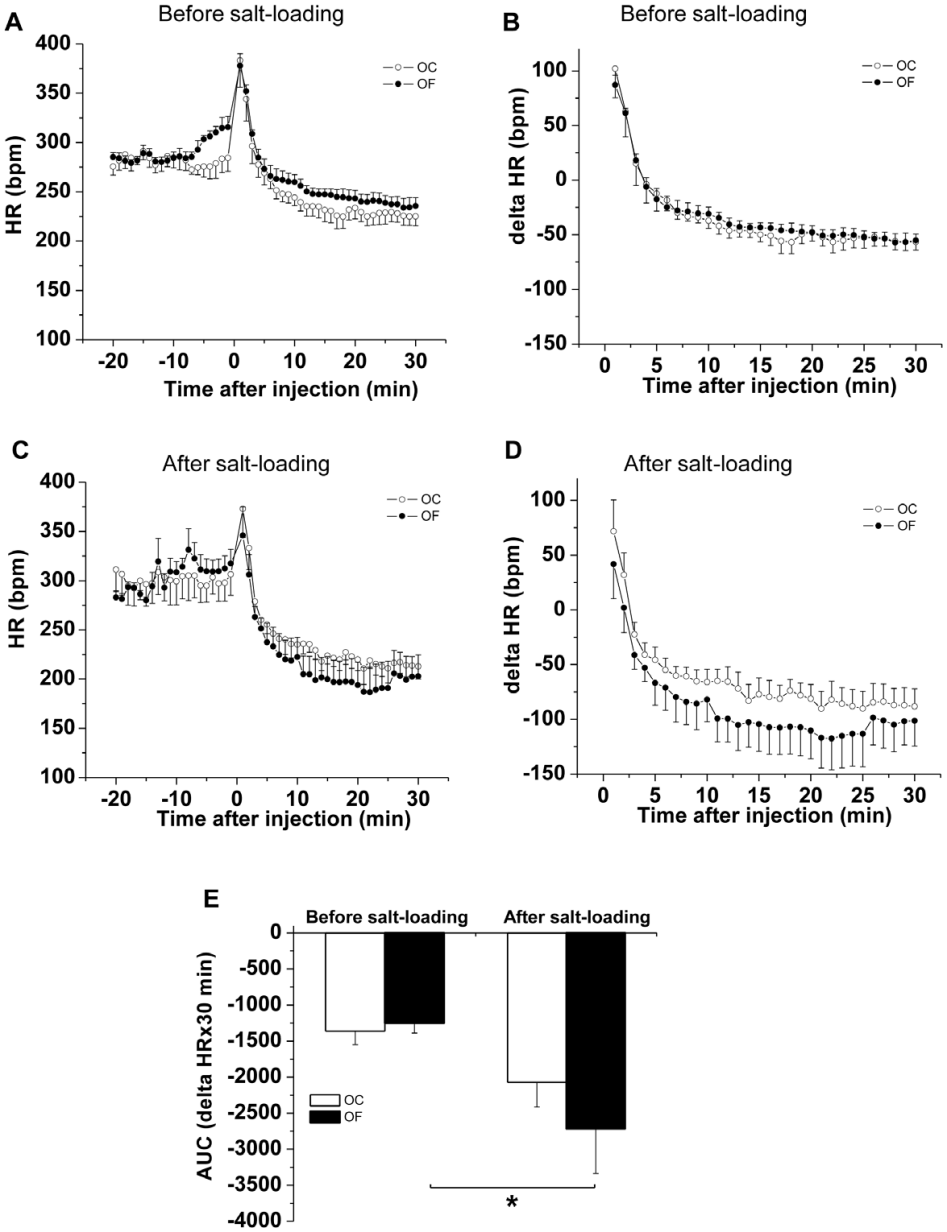
Male offspring of fat-fed dams had a greater bradycardiac response following adrenergic blockade after salt-loading than before. (A, C) HR response and delta HR (B, D) in response to combined adrenergic blockade before (A, B) and after (C, D) salt-loading in adult male offspring from dams fed a control (OC, open circles) or a fat diet (OF, closed circles). (E) Area under the curve of delta HR in response to combined adrenergic blockade before and after salt-loading in adult male offspring from dams fed a control (unfilled bars) or a fat diet (filled bars). n = 5 per group, * P≤0.05 versus before salt-loading. Error bars represent mean±SEM.

During salt-loading OF males exhibited elevated night-time (active phase) SBP (p = 0.04, n = 6) compared with OC males, which did not demonstrate a pressor response to the salt-load (Figure 4). Adrenergic receptor blockade after salt-loading (performed during the day) led to a greater fall in SBP and HR in OF males than before salt-loading (Figures 5 and 6). Blood pressure in female OC and OF was not influenced by salt-loading (Figure 4). During the week following salt loading cardiovascular parameters returned to basal values in male OF.

Salt-loading was associated with an increase from baseline in the low frequency∶high frequency (LF/HF) ratio of power spectral density of the heart rate in OF males (mean±SEM (SD), OC: 0.37±0. 07 (0.17) *versus* OF: 0.65±0.11 (0.27), p = 0.05, n = 6) although LF and HF spectral bands were comparable. There were no differences in LF/HF between OC and OF females following salt-loading. Also there was no effect of salt-loading on BPV power spectrum in OC and OF in either sex (data not shown).

## Discussion

The origins of hypertension are found in the interaction between genes and the environment. While the genetic variants contributing to the heritable traits of raised blood pressure have yet to be definitively described, the principal environmental causes are considered to be stress [14], dietary salt intake [15] and diet-induced obesity [16], [17]. Based on the data from the rodent model described here, we now propose that a maternal diet rich in animal fats adversely influences the developing offspring, leading to a lifelong predisposition towards exaggerated responses to two of these environmental determinants, stress and dietary salt. As this occurred in the absence of maternal obesity, it is likely to be directly related to components of the diet. Although the independent effect of a maternal fat-rich diet on cardiovascular and autonomic function in adult offspring has seldom been investigated [18] this study adds to a growing body of literature which reports adverse consequences of prenatal fat feeding [18], [19], [20] including the demonstration of glucose intolerance in weanling offspring of fat fed rats [19] and a recent study identifying increased risk of behavioural disorders in young offspring of fat fed macaques [20]. Our study therefore provides further support for the ‘developmental origins of disease’ hypothesis, which proposes that early life nutritional status may make a significant contribution to cardiovascular risk in adulthood. We have also confirmed, and expanded upon our previous observations of a central role for increased sympathoexcitation implicating abnormalities in autonomic control of blood pressure in offspring of rodents fed hypercalorific diets [8].

We hypothesised that the sympathetic over-activation and pressor responses observed in offspring of obese dams which we reported recently were, at least in part, mediated by the direct influence of the fat component of the obesogenic diet [8]. Our primary hypothesis was confirmed since the offspring of the fat fed dams, despite an absence of maternal weight gain, showed increased autonomic responsiveness to the challenges of acute stress and salt-loading. Others have reported effects of early life influences on aberrant autonomic control of offspring cardiovascular function. Indeed, the diversity of maternal interventions which affect abnormalities in this pathway implies a high degree of developmental plasticity and vulnerability in the relevant neuronal circuitry [21]. In rats, prenatal stress [22], hypoxia [23] and glucocorticoid exposure [24], malnutrition [25], salt-loading [26] and maternal obesity [8] all increase pressor responsiveness to an acute stress in adult offspring. Prenatal glucocorticoid exposure may also play a role in a model of maternal fat feeding as we have previously reported two-fold increase in corticosterone in plasma of high-fat fed dams (day 20 of gestation) compared to controls [27]. Furthermore, severe maternal protein restriction [28] and diabetes [29] in pregnancy induce salt-sensitive hypertension in the progeny. Intrauterine growth restriction induced by bilateral uterine artery ligation has also been found to increase systolic and diastolic BPV in aged rats [30] and exposure to prenatal hypoxia leads to altered HRV and BPV in response to acute stress, but normal basal values as reported in the present study [23]. Whether a shared mechanism is involved remains to be determined, although all interventions may increase oxidative and inflammatory signalling in the brain [31], [32] and thereby alter DNA environment [31]. Indeed by feeding fat fed dams antioxidants others have suggested that oxidative stress contributes to development of hypertension in the offspring of mice fed a high-fat diet (60% total fat) over a prolonged period [33].

Using a similar protocol we have previously reported raised baseline blood pressure in 6 and 12 month-old female offspring of fat fed dams [34]. The major difference between the present study and the previous report from our laboratory was the significant maternal weight gain observed in the previous study [34], strengthening the conclusion that the elevated basal blood pressure previously reported in offspring of obese [8] and fat fed dams [34] is a function of increased maternal adiposity rather than the fat content *per se*, although a direct effect of the high-sugar content of the obesogenic diet cannot be excluded. Others have reported that offspring of non-obese rats fed a high n-6/n3 PUFA diet demonstrate increased basal SBP [18]. However blood pressure was determined by the tail-cuff method, which is recognised to evoke an acute stress response of itself [25]. Variation in the degree of maternal weight gain in response to the high fat diet in different studies could be explained by the strain of rat studied in as much as Sprague-Dawley rats appear to contain two separate phenotypes with regard to their propensity to develop diet-induced obesity [35].

To further address the importance of maternal weight gain in determination of offspring resting SBP, we performed an exploratory correlation analysis (using Pearson's product-moment correlation) between maternal weight gain and offspring mean SBP and found a positive association independent of maternal diet (day-time, resting phase SBP: r = 0.543, r^2^ = 0.295 (95% CI for r^2^ 0.025 to 0.616, p = 0.009); night-time, active phase SBP: r = 0.536, r^2^ = 0.287 (95% CI for r^2^ 0.022 to 0.610), p = 0.010 n = 22). Interestingly, a human mother-child cohort study has similarly revealed a positive association between gestational weight gain and the offspring's blood pressure in adulthood [2].

The absence of resting hypertension in the offspring of the fat fed dams may be also be explicable on the basis of the relative leanness of the offspring compared to those in other studies in which hypertension coincided with increased offspring body weight [5], [8], [18], [33], [34,] adiposity [5], [8], and hyperleptinaemia [5]. In animal models of overnutrition, maternal weight gain and offspring adiposity therefore appear to be more important determinants of raised basal blood pressure than the fat component of the diet [8], [34]. Indeed female offspring of fat fed dams in the current study were hypo-leptinaemic compared to controls and this may confer cardiovascular “protection” as stimulation of renal sympathetic nerve activity via hyperleptinaemia may contribute to the elevated SBP [16], [36].

Interestingly, we observed elevated plasma noradrenaline in OF females associated with a two-fold increase in plasma FFA concentration and decreased leptin level. This is in agreement with other reports in which; food deprivation and cold exposure [37]; strenuous military training exercises [38]; in vivo administration of isoprenaline [39]; and dopamine D2 receptor agonist bromocriptine [40] are shown to decrease plasma leptin in association with sympathetic nervous activation of adipose tissue, increased lipolysis and elevated FFA. It was suggested that the product of activated lipolysis, long-chain FFA, could be a metabolic signal mediating the inhibitory effect of noradrenaline on leptin secretion from adipocytes [41]. Despite pronounced hypoleptinaemia in OF females, the abdominal fat mass and BW were similar between the two groups; locomotor activity was not significantly increased and FFA concentration did not correlate with plasma leptin level in OF females.

Pharmacological blockade of the adrenergic pathways abolished the stress-induced changes in SBP in offspring of the fat fed dams thus implicating sympathoactivation in the stress-induced hypertension. Prolonged cardiovascular recovery after acute stress in male offspring of fat fed dams may involve increased stress-induced renal sympathetic nerve activity (SNA). Renal venous noradrenaline spill over would provide an accurate indirect measure of renal SNA, whilst direct nerve activity recording by radiotelemetry is also now possible. In addition, blunted endothelium-dependent relaxation of isolated resistance (mesenteric) vessels which we have reported previously in this model [34] due to impaired EDHF-mediated relaxation [42] may also contribute to prolonged stress recovery of SBP.

Lack of depressor response following adrenergic blockade in OC could be explained by predominant impact of other stress-related factors (i.e. HPA or RAAS) rather than sympathetic nerves. Stress-induced HR response was abolished after blockade in both OC and OF groups. Beta-adrenergic and muscarinic blockade in Wistar rats has shown that sympathetic activation is responsible for the sustained component of restraint stress-induced tachycardia whereas vagal withdraw contributes to the initial transient peak component [43].

Since the stress response is mediated not only by the sympatho-adrenomedullary axis but, also by the hypothalamo-pituitary-adrenocortical (HPA) axis, the persistent activation of the HPA pathway may also underlie the exacerbated stress-response in male offspring of fat fed dams. Several models of developmental programming demonstrate permanent alterations of the HPA axis in rats secondary to maternal undernutrition [44], [45], prenatal stress or dexamethasone exposure [46], [47]. The HPA pathway requires investigation in this model.

In man and animals the autonomic nervous system is implicated in salt-sensitive hypertension [48], [49]. Dysregulation of sodium homeostasis is proposed to increase the cerebrospinal fluid sodium concentration and heighten neuronal responsiveness [49] contributing to exaggerated sympathetic outflow [11]. In the present study, salt-loading resulted in hypertension in male offspring of fat fed dams during their nocturnal period of heightened activity. The increase of the LF/HF ratio of HRV in male offspring after salt-loading indicated a shift of sympatho-vagal balance at the sinus node towards sympathetic control, since LF power is modulated by both the sympathetic and parasympathetic nervous system [12] and HF power is parasympathetically mediated [12], [13]. Whilst the adrenergic component of the salt-sensitive hypertension shows only a modest increase as measured during the day, taken together with the increase in LF/HF ratio it provides evidence for exaggerated sympathetic tone after salt-loading in offspring of fat fed dams. The effect of adrenergic blockade after salt loading if measured at night-time would also likely to be more pronounced when the animals are active, and blood pressure was elevated above controls. Notwithstanding the limitations in the indirect non-invasive technique (reviewed in [50]) the results obtained from HRV analysis are consistent with the other cardiovascular parameters reported. Direct recordings of the renal SNA by radiotelemetry, before and after salt-loading in both groups would confirm changes in the autonomic control of the blood pressure. Our data is consistent with other reports in rodents implicating sympathoactivation in salt-sensitive hypertension [11], [15]. Dysfunctional renal nephron development is unlikely to contribute as we previously showed no differences in glomerular number or volume in offspring of fat fed dams compared to controls [51]. Sprague-Dawley rats are not intrinsically salt-sensitive [52] which may explain the lack of the pressor response to salt-loading in the control animals. Further interrogation of parameters of the renin-angiotensin system before and after salt-loading may provide further insight into differential salt sensitivity between the offspring of control and fat fed dams.

Observed autonomic changes were limited predominantly to male offspring since female offspring of fat fed dams exhibited similar cardiovascular responses to acute stress and salt-load as controls. Sex differences in developmental programming of cardiovascular dysfunction have frequently been reported but are poorly understood [6], [34], [53]. The predominance of the male phenotype in the current study accords with other models [5]–[8], [53] perhaps because females are more likely to have estrogen associated protection from development of cardiovascular disorders [54]. The mechanisms of sexual dimorphism in developmental programming may involve interaction between the early-life intervention and the sex hormones [55], or the sex chromosomes, and reversible epigenetic modifications such as histone acetylation and methylation status of promoter/enhancer regions of certain genes have been implicated [56]. It may be of relevance that the Sry locus which is an evolutionarily conserved locus on the mammalian Y chromosome has been implicated in sex differences in blood pressure regulation, sympathetic nervous system, renin–angiotensin system and androgen receptor regulation (reviewed in [57]). However, the interaction between maternal diet and offspring sex is clearly complex and beyond the scope of this study.

The lower DBP and MAP in females versus male offspring are well recognized [58], [59]. The underlying mechanisms may include sex hormones [56], different distribution of AT_1_ receptors [60] or the Y chromosome [57]. In agreement with a previous report, control females had a higher HR and, correspondingly, smaller mean IBI of HRV than males [58]. Higher HR was also present in female offspring of fat fed dams too and associated with greater locomotor activity compared with males born to fat fed dams.

In conclusion, we have demonstrated that short-term exposure to diet rich in lard in rat dams, in the absence of overt maternal obesity, is not associated with elevation of resting blood pressure but adversely influences autonomic pathways regulating cardiovascular control and results in increased cardiovascular reactivity to acute stress and salt-loading in the male offspring. These data infer developmental plasticity of the sympathetic nervous system to maternal dietary components. We therefore propose that maternal fat feeding may contribute to increased risk of offspring hypertension in response to established adulthood environmental factors through dysfunction of the autonomic nervous system.

This study supports the premise that maternal dietary animal fats make a significant contribution to blood pressure dysregulation and risk of cardiovascular disease in adulthood. Should this observation pertain to women and their children, reduction of animal fats in the diet of pregnant women may reduce the risk of cardiovascular disease in adulthood.

## Materials and Methods

### Experimental diet dams and litters

All procedures were performed in accordance with the UK Home Office Guidance on the Operation of the Animals (Scientific Procedures) Act 1986 (UK Home Office. Project Licence No. PPL 70/7090). Female Sprague-Dawley rats (100±10 days old, n = 27) (Charles River Laboratories, UK) were fed either a control diet of standard chow (10.0% moisture, 4.3% fat, 22.4% protein, 55.7% carbohydrates, 7.6% ash, vitamins and minerals, 15.2 MJ/kg, RM3, Special Diet Services) or an experimental fat-rich diet enriched with animal lard (8.2% moisture, 23.6% fat, 21.6% protein, 41.1% carbohydrates, 5.5% ash, vitamins and minerals, 19.6 MJ/kg) for 10 days pre-conception, throughout mating, gestation and lactation (dietary fatty acid components are presented in Table 1). Rats were caged separately (20°C and 60% humidity; light-dark cycle 12 hours) and food and water given *ad libitum*. At 2 days post-partum all litters were limited to 4 males and 4 females (when possible) to standardize milk availability during suckling. At 21 days (weaning) offspring of control (OC) and fat fed (OF) dams were weaned onto a standard chow diet (RM1, Special Diet Services) and housed by sex. The non-fasting dams were sacrificed by a rising concentration of CO_2_ in a euthanasia chamber (day 21) and blood samples obtained by cardiac puncture for measurement of leptin and insulin. One male and 1 female from each litter were similarly sacrificed at 9 months and blood samples and kidney tissue obtained; the remaining littermates (1 male and 1 female from each litter) were utilized for evaluation of cardiovascular reactivity at 3, 6 and 9 months.

### Radiotelemetry

Systolic blood pressure (SBP), diastolic blood pressure (DBP), mean arterial pressure (MAP), HR and locomotor activity were measured in the offspring by radio-telemetry as described previously, in randomly selected time matched littermates (1 male and 1 female from each litter) [34]. Full details of the methodology are given in Methods S1.

### Cardiovascular Responses to Acute Stress and Adrenergic Receptor Blockade

Humane Perspex whole body restraint cylinders were employed to immobilise OC and OF rats and to induce stress. The size of the cylinder was selected according to sex and body weight (BW). Cardiovascular parameters were recorded in animals for 10 sec every minute during 30 min of restraint and 120 min after return to the home cage. Experiments were performed between 0900 and 1200hrs in matched paired animals after establishing baseline recordings between 0700 and 0830hrs. In a sub-group of animals, a combination of the non-selective beta-adrenergic receptor antagonist propranolol, and the alpha-1-adrenergic receptor antagonist terazosin (both drugs 10 mg/kg BW in saline; Sigma-Aldrich Ltd., Poole, UK) was injected 1 hour prior to the second stress test (intraperitoneal i.p.). Receptor blockade was not verified in these protocols, but assumed from satisfactory blockade achieved with these doses in previous studies [61], [62]. The second stress-test (total adrenergic blockade) was performed two days after the first stress (baseline).

### Cardiovascular Response to Salt-Loading

To assess response to salt-loading, telemetred animals were fed a commercial high-salt diet (RM1 supplemented with 8%NaCl, SDS, UK) *ad libitum* for 1 week and then fed RM1 for 1 week (0.8%NaCl). Cardiovascular variables were recorded for 10 sec every 5 min during a 1 week baseline period, during salt-loading and for 1 week thereafter. Adrenergic receptor blockade was performed before and after salt-loading in male offspring only.

### Heart Rate and Blood Pressure Variability

HRV and systolic BPV were analysed from a 300 sec continuous telemetric blood pressure record made between 0900 and 1000 hrs in undisturbed telemetred animals in a quiet room. Data sets recorded in a sinus rhythm with sampling frequency 500 Hz were used. Time and frequency domain of HRV analysis were performed using HRV module of Chart 5.0 analysing software (ADInstruments, Colorado Springs, CO). Spectral powers of blood pressure signals were analysed with the LabVIEW 7.1 (National Instruments, USA) programming environment, which has built-in methods for spectral analysis. Integrated boundaries for spectral bands were set at 0.2–0.6 Hz for low frequency (LF) and 0.6–2.5 Hz for high-frequency (HF) component [63]. Full details of the methodology are given in Methods S1.

### Baroreceptor Function

Baroreceptor function was assessed as described previously (22) and adapted to telemetred rats. Full details of the methodology are given in Methods S1.

### Plasma Analysis

Animals were sacrificed by a rising concentration of CO_2_ in a euthanasia chamber. Maternal and adult offspring blood samples were collected post mortem by cardiac puncture. Plasma were stored at −80°C prior to analysis. Plasma leptin concentration was analysed by ELISA according to manufacturer's instruction (RD1991 kit; Biovendor, Modrice, Czech Rep.) as was insulin (nr.17700; Mercodia, Uppsala, Sweden). FFA concentrations were determined in plasma extracts by autoanalyser (Synchron LX-20 Pro Autoanalyser, Beckman Coulter Inc., Woerden, The Netherlands) using commercial kit (NEFA-C from Wako Chemicals, Neuss, Germany). Plasma noradrenaline contents were determined in offspring plasma using the EIA assay kit (ALPCO Diagnostics, Salem, USA) according to the manufacturer's protocol.

### Statistical Analysis

All results are presented as mean±SEM with standard deviations given for the main outcome measures as an indication of variability. For all experimental parameters reported 1 male and 1 female from each litter were recorded. Therefore the statistical unit is the maternal environment where ‘n’ refers to the number of dams in a given group. For most parameters comparisons were made using unpaired two-tailed Student's t-test (significance p≤0.05). Based on previous observations of cardiovascular parameters, males and females were analysed separately. Standard checks for normality, skewness and kurtosis were performed. Responses to stresses were expressed as a percentage change from baseline for the periods of restraint and recovery and compared by RM ANOVA followed by Bonferroni post-hoc test. Salt-loading data represents diurnal averages for 7 days analysed by RM ANOVA. Analyses were performed with GraphPad Prism5 unless otherwise stated.

## Supporting Information

Figure S1 **Male offspring of fat-fed dams had an increased pressor response during acute stress.** (A–C) DBP and (D–F) MAP responses during 30 min of acute stress and 120 min of recovery, before (A, D) and after adrenergic receptor blockade (B, E) in male offspring born to dams fed a control (open circles) or a fat diet (closed circles), n = 5–11 per group. *P≤0.05, **P≤0.01 *versus* control. (C) Area under the curve for DBP and (F) MAP during stress before and after blockade in male offspring born to dams fed a control (unfilled bars) or a fat diet (filled bars). n = 5–11 per group. *P≤0.05 *versus* control, †††P≤0.001 versus before blockade. Error bars represent mean±SEM.
(TIF)

Figure S2 **Female offspring of fat-fed dams had a pressor response during acute stress.** (A–C) DBP and (D–F) MAP responses during 30 min of acute stress and 120 min of recovery, before (A, D) and after adrenergic receptor blockade (B, E) in female offspring born to dams fed a control (open circles) or a fat diet (closed circles), n = 5–15 per group. (C) Area under the curve for DBP and (F) MAP during stress before and after blockade. *P≤0.05 *versus* before blockade in female offspring born to dams fed a control (unfilled bars) or a fat diet (filled bars). Error bars represent mean±SEM.
(TIF)

Figure S3 **Male and female offspring of fat-fed dams had an increased pressor response during acute stress at 3 month of age.** (A, C) SBP and (B, D) HR responses during 30 min of acute stress and 120 min of recovery in male (A, B) and female (C, D) offspring born to dams fed a control (open circles) or a fat diet (closed circles), n = 6 per group for males and n = 7 per group for females. *P≤0.05, **P≤0.01 *versus* control. Error bars represent mean±SEM.
(TIF)

Figure S4 **Male offspring of fat-fed dams had an increased pressor and chronotropic response during acute stress at 6 month of age.** (A, C) SBP and (B, D) HR responses during 30 min of acute stress and 120 min of recovery in male (A, B) and female (C. D) offspring born to dams fed a control (open circles) or a fat diet (closed circles), n = 6 per group for males and n = 7 per group for females. *P≤0.05, **P≤0.01 *versus* control. Error bars represent mean±SEM.
(TIF)

Table S1 **Basal cardiovascular parameters and activity in 3 month old offspring born to dams fed a control (OC) or a fat diet (OF).** Data given as HR, mean±SEM (SD).
(PDF)

Table S2 **Basal cardiovascular parameters and activity in 6 month old offspring born to dams fed a control (OC) or a fat diet (OF).** Data given as HR, mean±SEM (SD).
(PDF)

Methods S1 **Supplementary methods.**
(PDF)
