# Supplementary figures and images for: Increased Cardiovascular Reactivity to Acute Stress and Salt-Loading in Adult Male Offspring of Fat Fed Non-Obese Rats
Source: PLoS One. 2011 Oct 17;6(10):e25250. doi: 10.1371/journal.pone.0025250 (PMC3197190; doi:10.1371/journal.pone.0025250)

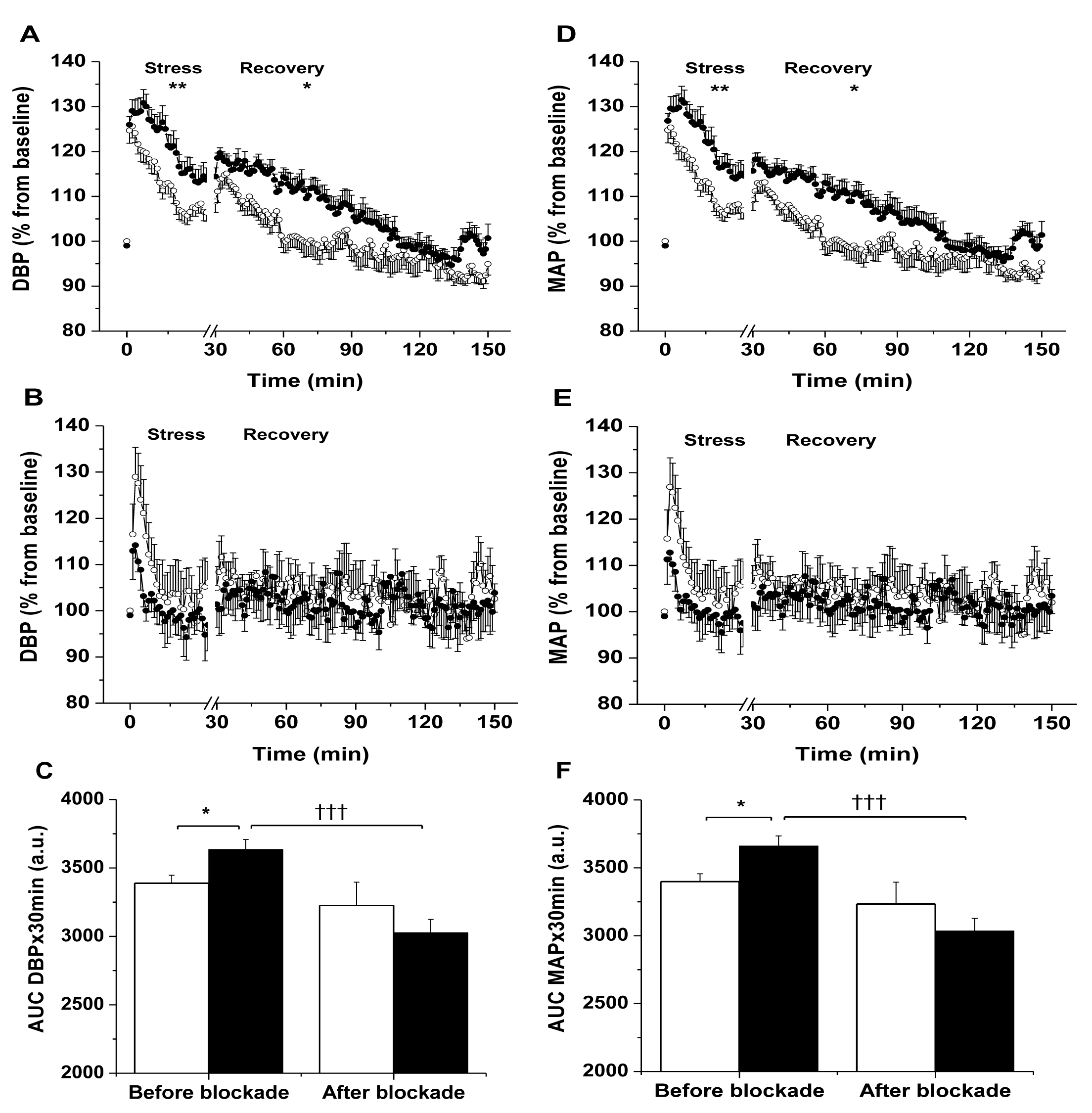

Supplement: Figure S1 — Male offspring of fat-fed dams had an increased pressor response during acute stress. (A–C) DBP and (D–F) MAP responses during 30 min of acute stress and 120 min of recovery, before (A, D) and after adrenergic receptor blockade (B, E) in male offspring born to dams fed a control (open circles) or a fat diet (closed circles), n = 5–11 per group. *P≤0.05, **P≤0.01 versus control. (C) Area under the curve for DBP and (F) MAP during stress before and after blockade in male offspring born to dams fed a control (unfilled bars) or a fat diet (filled bars). n = 5–11 per group. *P≤0.05 versus control, †††P≤0.001 versus before blockade. Error bars represent mean±SEM. (TIF) [file pone.0025250.s001.tif]

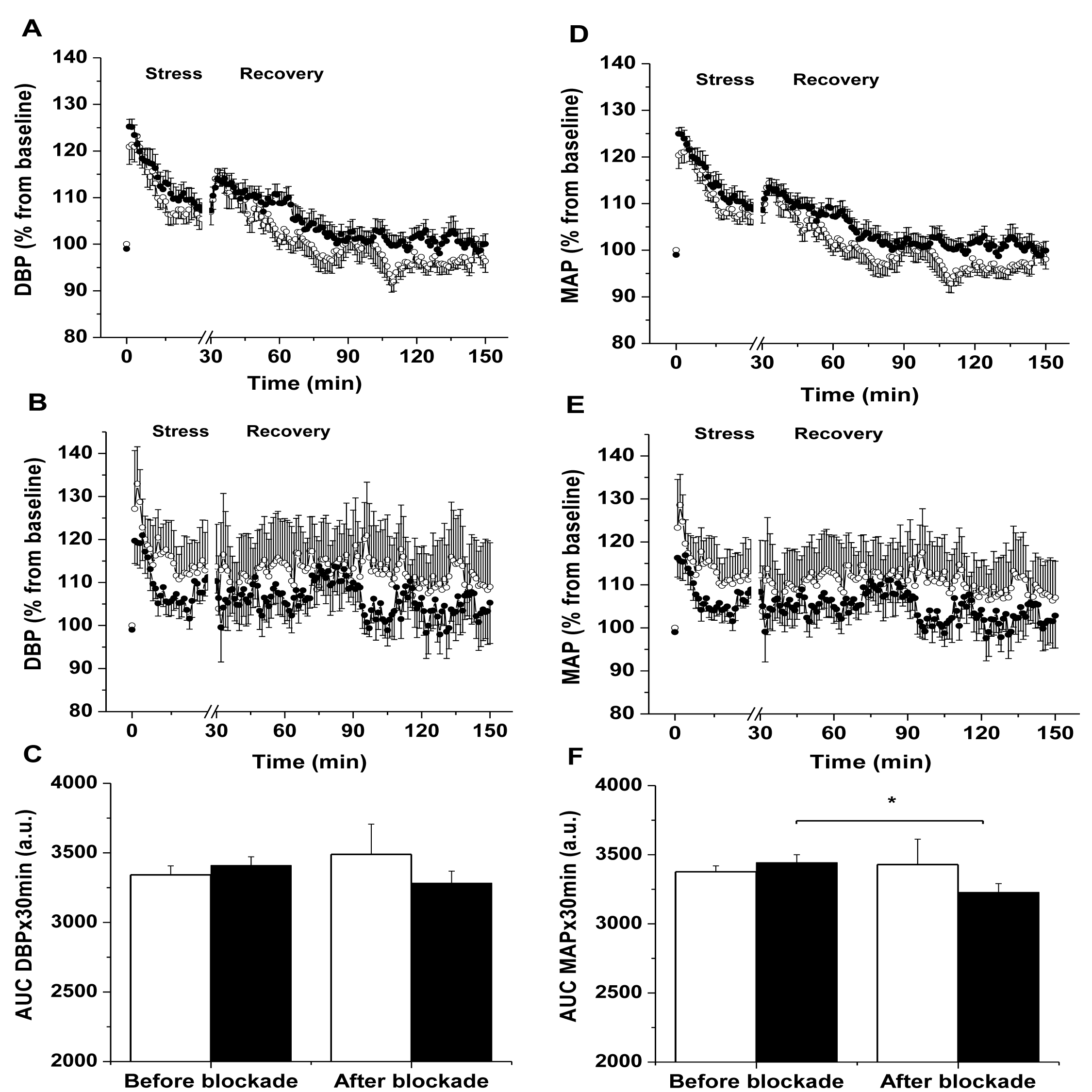

Supplement: Figure S2 — Female offspring of fat-fed dams had a pressor response during acute stress. (A–C) DBP and (D–F) MAP responses during 30 min of acute stress and 120 min of recovery, before (A, D) and after adrenergic receptor blockade (B, E) in female offspring born to dams fed a control (open circles) or a fat diet (closed circles), n = 5–15 per group. (C) Area under the curve for DBP and (F) MAP during stress before and after blockade. *P≤0.05 versus before blockade in female offspring born to dams fed a control (unfilled bars) or a fat diet (filled bars). Error bars represent mean±SEM. (TIF) [file pone.0025250.s002.tif]

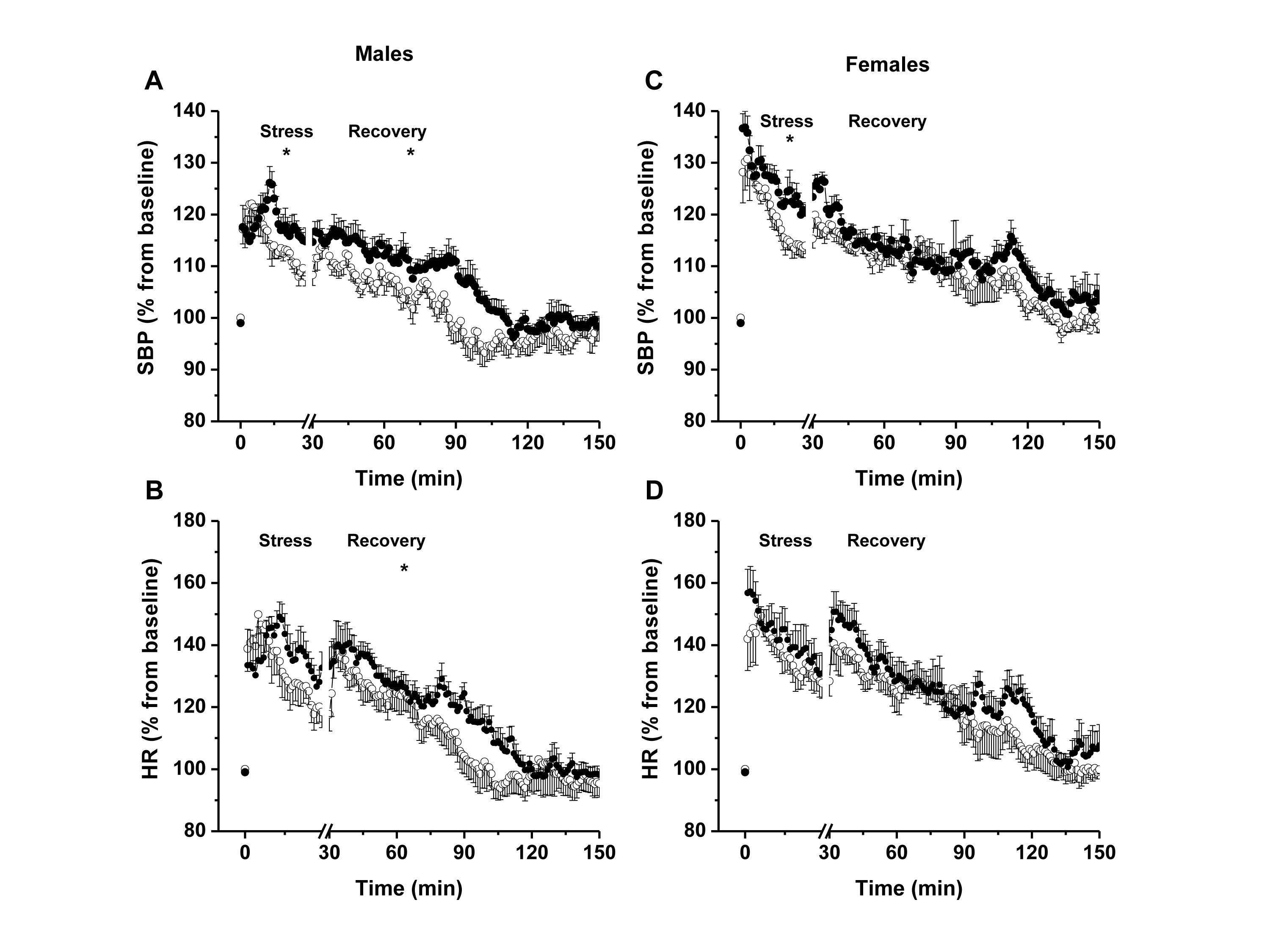

Supplement: Figure S3 — Male and female offspring of fat-fed dams had an increased pressor response during acute stress at 3 month of age. (A, C) SBP and (B, D) HR responses during 30 min of acute stress and 120 min of recovery in male (A, B) and female (C, D) offspring born to dams fed a control (open circles) or a fat diet (closed circles), n = 6 per group for males and n = 7 per group for females. *P≤0.05, **P≤0.01 versus control. Error bars represent mean±SEM. (TIF) [file pone.0025250.s003.tif]

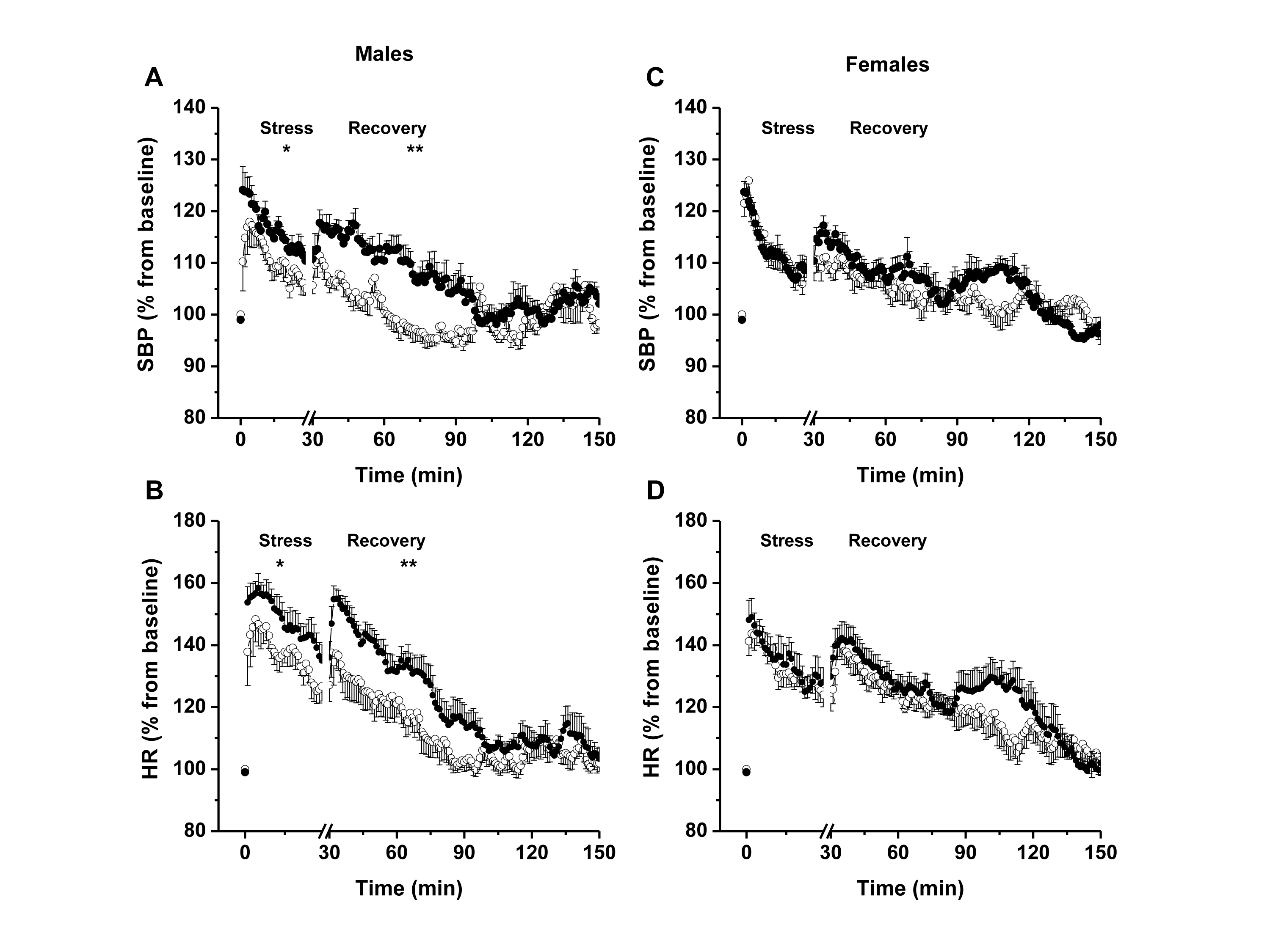

Supplement: Figure S4 — Male offspring of fat-fed dams had an increased pressor and chronotropic response during acute stress at 6 month of age. (A, C) SBP and (B, D) HR responses during 30 min of acute stress and 120 min of recovery in male (A, B) and female (C. D) offspring born to dams fed a control (open circles) or a fat diet (closed circles), n = 6 per group for males and n = 7 per group for females. *P≤0.05, **P≤0.01 versus control. Error bars represent mean±SEM. (TIF) [file pone.0025250.s004.tif]
