# Supplementary material for: Increased Cardiovascular Reactivity to Acute Stress and Salt-Loading in Adult Male Offspring of Fat Fed Non-Obese Rats
Source: PLoS One. 2011 Oct 17;6(10):e25250. doi: 10.1371/journal.pone.0025250 (PMC3197190; doi:10.1371/journal.pone.0025250)
Supplement: Table S1 — Basal cardiovascular parameters and activity in 3 month old offspring born to dams fed a control (OC) or a fat diet (OF). Data given as HR, mean±SEM (SD). (PDF) [file pone.0025250.s005.pdf]

Table S1. Basal cardiovascular parameters and activity in 3 month old offspring born to dams fed a control (OC) or a fat diet (OF). Data given as mean±SEM (SD). †† P≤0.01 *versus* offspring of the same dietary group.

	OC (n=7)	OF (n=8)	OC (n=7)	OF (n=8)
	Males		Females	
Day Systolic blood pressure (mmHg)	115.7±3.5 (9.3)	121.6±4.9 (13.9)	123.1±3.2 (8.5)	123.4±2.6 (7.4)
Night Systolic blood pressure (mmHg)	125.5±5.2 (13.8)	125.8±4.9 (13.9)	126.9±2.8 (7.4)	127.4±2.5 (7.1)
Day Diastolic blood pressure (mmHg)	86.8±3.1 (8.2)	85.2±2.7 (7.6)	85.6±2.4 (6.4)	83.9±1.4 (4.0)
Night Diastolic blood pressure (mmHg)	89.4±3.2 (8.5)	88.7±2.5 (7.1)	88.6±2.2 (5.8)	87.4±1.4 (4.0)
Day Heart Rate (bpm)	350±9 (23.8)	342±6 (17.0)	388±7 (19) ††	370±6 (17) ††
Night Heart Rate (bpm)	375±9 (23.8)	368±7 (19.8)	417±7 (19) ††	399±5 (14) ††
Day Mean arterial pressure (mmHg)	102.3±3.5 (9.3)	101.2±3.4 (9.6)	102.6±2.9 (7.7)	100.0±2.9 (8.2)
Night Mean arterial pressure (mmHg)	105.7±3.8 (10.1)	104.9±3.2 (9.1)	105.9±2.6 (6.9)	105.6±1.8 (5.1)
Day Activity (counts per min)	2.3±0.3 (0.8)	2.5±0.2 (0.6)	2.5±0.2 (0.5)	2.50±0.3 (0.9)
Night Activity (counts per min)	4.0±0.5 (1.3)	2.1±0.3 (0.9)	4.1±0.2 (0.5)	4.2±0.5 (1.4) ††

Data given as mean±SEM (SD). †† $P \leq 0.01$ *versus* offspring of the same dietary group.
