# Supplementary material for: Increased Cardiovascular Reactivity to Acute Stress and Salt-Loading in Adult Male Offspring of Fat Fed Non-Obese Rats
Source: PLoS One. 2011 Oct 17;6(10):e25250. doi: 10.1371/journal.pone.0025250 (PMC3197190; doi:10.1371/journal.pone.0025250)
Supplement: Table S2 — Basal cardiovascular parameters and activity in 6 month old offspring born to dams fed a control (OC) or a fat diet (OF). Data given as HR, mean±SEM (SD). (PDF) [file pone.0025250.s006.pdf]

Table S2. Basal cardiovascular parameters and activity in 6 month old offspring born to dams fed a control (OC) or a fat diet (OF). Data given as mean±SEM (SD). †† P≤0.01 *versus* offspring of the same dietary group.

	OC (n=6)	OF (n=7)	OC (n=7)	OF (n=7)
	Males		Females	
Day Systolic blood pressure (mmHg)	129.2±0.5 (1.2)	129.8±0.3 (0.8)	123.2±0.5 (1.3) ††	120.6±0.5 (1.3) ††
Night Systolic blood pressure (mmHg)	133.5±0.5 (1.2)	135.9±0.5 (1.3)	128.5±0.4 (1.1) ††	126.2±0.4 (1.1) ††
Day Diastolic blood pressure (mmHg)	89.5±0.3 (0.7)	86.2±0.4 (1.1)	82.5±0.5 (1.3) ††	83.8±0.5 (1.3) ††
Night Diastolic blood pressure (mmHg)	94.5±0.4 (1.0)	93.2±0.6 (1.6)	87.9±0.3 (0.8) ††	89.6±0.4 (1.1) ††
Day Heart Rate (bpm)	352±3 (7)	342±3 (8)	373±4 (11) ††	374±3 (8) ††
Night Heart Rate (bpm)	404±3 (7)	398±4 (11)	422±3 (8) ††	433±3 (8) ††
Day Mean arterial pressure (mmHg)	106.9±0.3 (0.7)	106.0±0.3 (0.8)	100.8±0.5 (1.3) ††	100.1±0.5 (1.3)
†† Night Mean arterial pressure (mmHg)	111.5±0.5 (1.2)	111.5±0.6 (1.6)	106.1±0.4 (1.1) ††	106.1±0.4 (1.1) ††
Day Activity (counts per min)	1.7±0.2 (0.5)	1.3±0.1 (0.3)	1.7±0.2 (0.5)	1.8±0.1 (0.3) ††
Night Activity (counts per min)	4.4±0.3 (0.7)	4.1±0.3 (0.8)	4.5±0.2 (0.5)	5.6±0.2 (0.5) ††

Data given as mean±SEM (SD). †† P≤0.01 *versus* offspring of the same dietary group.
