# Supplementary material for: Increased Cardiovascular Reactivity to Acute Stress and Salt-Loading in Adult Male Offspring of Fat Fed Non-Obese Rats
Source: PLoS One. 2011 Oct 17;6(10):e25250. doi: 10.1371/journal.pone.0025250 (PMC3197190; doi:10.1371/journal.pone.0025250)
Supplement: Methods S1 — Supplementary methods. (PDF) [file pone.0025250.s007.pdf]

SUPPLEMENTAL MATERIAL

Increased cardiovascular reactivity to acute stress and salt-loading in adult male offspring of fat fed non-obese rats

Olena Rudyk[1, 2], Péter Makra[3], Eugene Jansen[4], Michael J. Shattock[2], Lucilla Poston[1] and Paul D. Taylor[1]

[1] Division of Women's Health in the School of Medicine, [2]Cardiovascular Division, King's College London, London UK, [3]Department of Experimental Physics, University of Szeged, Hungary, [4]Laboratory for Health Protection Research, National Institute for Public Health and the Environment, Bilthoven, the Netherlands.

Corresponding author:

Dr Paul Taylor

Division of Women's Health

King's College School of Medicine,

10th Floor North Wing

St. Thomas' Hospital

London SE1 7EH, UK

Tel. 020 7188 3630, Fax. 020 7188 1227

E-mail: pail.taylor@kcl.ac.uk

Supplementary Methods

Radiotelemetry.

Randomly selected time matched littermates at 9 month of age were anesthetised with 2% isofluorane in O_2 at 2l per minute with pre and post-operative analgesia (buprenorphine, 0.1mg/kg). Following routine laparotomy, the catheter of the PhysioTel®PA-C40 pressure transmitter (Data Science International) was surgically implanted into the descending abdominal aorta and the body of the probe transfixed to the abdominal wall. Following one week of recovery, rats housed in individual cages were placed above the telemetric receivers with output to a computer. Cardiovascular variables were routinely monitored and recorded by scheduled sampling for 10sec every 5min (sampling frequency 500Hz) with A.R.T. (Dataquest IV, DSI) software.

Heart Rate and Blood Pressure Variability.

Heart Rate and systolic Blood Pressure Variability were analysed from a 300sec continuous telemetric blood pressure record made between 0900 and 1000hrs in undisturbed telemetred animals in a quiet room. Data sets recorded in a sinus rhythm with sampling frequency 500Hz were used. Time and frequency domain of HRV analysis were performed using HRV module of Chart 5.0 analysing software (ADInstruments, Colorado Springs, CO). Ectopics and visible short artefacts were manually excluded or replaced by intervals linearly interpolated from the nearest normal interval in order to avoid discontinuity in the record. Integrated boundaries for spectral bands were set at 0.2-0.6Hz for low frequency (LF) and 0.6-2.5Hz for high-frequency (HF) component.

Spectral powers of blood pressure signals were analysed with the LabVIEW 7.1 (National Instruments, USA) programming environment, which has built-in methods for spectral

analysis. First, the mean value from the SBP time sequence was subtracted to eliminate the DC component. No additional low-pass filtering was performed, since the anti-aliasing filter in the data recording equipment ensures that no high-frequency contamination can appear in the relevant sampling frequency. Likewise, the original sampling frequency (500Hz) of the recording was used and data was not re-sampled at a lower frequency as the speed of the offline evaluation was not critical. The power spectral density of the resultant signal was calculated using a Hanning window, and the LF and HF spectral powers were obtained as follows:

$$\mathrm{LF} = \Delta f \sum_{j=j_{\min}}^{j_{\max}-1} S(j \cdot \Delta f),\ \ \mathrm{HF} = \Delta f \sum_{k=k_{\min}}^{k_{\max}-1} S(k \cdot \Delta f),$$

where Δf denotes the frequency resolution in the discrete spectrum and S stands for the power spectral density of the blood pressure. The indices $i_{\min}, i_{\max}, j_{\min}, j_{\max}, k_{\min}, k_{\max}$ correspond to the boundaries of the very low, low and high frequency ranges:

$$i_{\min} \cdot \Delta f = 0\ \mathrm{Hz};\ i_{\max} \cdot \Delta f = 0.2\ \mathrm{Hz} = j_{\min} \cdot \Delta f;\ j_{\max} \cdot \Delta f = 0.6\ \mathrm{Hz} = k_{\min} \cdot \Delta f;$$
$$k_{\max} \cdot \Delta f = 2.5\ \mathrm{Hz}.$$

The block size was about 150 000 (300sec), which ensures a frequency resolution of about Δf =0.0333Hz. The block size was allowed to vary slightly between recordings (between 148 500 and 150 000), which did not affect precision in determining the spectral power since a real frequency scale was used, not bins, thus the indices above were always precisely tailored to the individual recordings.

Baroreceptor Function.

Telemetred animals were anesthetised with 2% isofluorane and PE-50 catheter filled with heparinised saline (100Uml^{-1}) was introduced into left jugular vein, passed subcutaneously and exteriorized at the back of neck where it was secured and plugged. On recovery, rats were placed above the telemetric receivers. On the next day the catheter was flushed with heparinised saline and continuous monitoring of blood pressure was started in freely moving rats. 30min of baseline were recorded before baroreflex function was assessed by recording the maximal HR changes at the time of maximum increase and decrease of MAP induced by intravenous bolus injections of phenylephrine (PE; 1, 2 and 4µgkg^{-1}) or sodium nitroprusside (SNP; 5, 10 and 20µgkg^{-1}). Subsequent doses of PE or SNP were injected at increasing concentrations after the MAP returned to baseline values. All data were fitted to a sigmoid curve using non-linear regression with GraphPad Prism5 (GraphPad Software San-Diego, Ca, USA) software and the baroreflex gain at any given MAP was calculated from the first derivative of the sigmoid function. For the sigmoid regression curve analysis, top, bottom of the curve, logEC$_{50}$ and Hill slope were employed as logistic parameters.
